# Effect of Tungsten Oxide Nanostructures on Sensitivity and Selectivity of Pollution Gases

**DOI:** 10.3390/s20174801

**Published:** 2020-08-26

**Authors:** Fenghui An, Andrew F. Zhou, Peter X. Feng

**Affiliations:** 1 School of Mechanical and Materials Engineering, Jiujiang University, Jiujiang 332005, China; 2 Department of Physics, University of Puerto Rico, San Juan, PR 00936, USA; 3 Department of Physics, Indiana University of Pennsylvania, Indiana, PA 15705, USA; fzhou@iup.edu

**Keywords:** tungsten trioxide, surface morphology, gas sensor, hot-filament CVD

## Abstract

We report on the different surface structures of tungsten oxides which have been synthesized using a simple post-annealing-free hot-filament CVD technique, including 0D nanoparticles (NPs), 1D nanorods (NRs), and 2D nanosheet assemblies of 3D hierarchical nanoflowers (NFs). The surface morphologies, crystalline structures, and material compositions have been characterized by scanning electron microscopy (SEM), energy dispersive X-ray spectroscopy (EDS), X-ray diffraction (XRD), and Raman spectroscopy, respectively. The sensor performances based on the synthesized samples of various surface morphologies have been investigated, as well as the influences of operating temperature and applied bias. The sensing property depends closely on the surface morphology, and the 3D hierarchical nanoflowers-based gas sensor offers the best sensitivity and fastest response time to NH_3_ and CH_3_ gases when operated at room temperature.

## 1. Introduction

Gas-sensing technology has been employed extensively for environmental, industrial, and medical applications. The development of sensitive and reliable materials that detect small amounts of harmful contaminants in the air has been and remains a major challenge [1,2]. Various metal oxide semiconductors (MOS), used as conventional chemical gas-sensing materials in resistive type gas sensors, have been increasingly investigated because of their operational simplicity, lightweight, high sensitivity, and low cost [3,4,5]. Among these oxides, tungsten oxide thin films happen to be one of the promising candidates for sensing hazardous gases. Hence, a variety of tungsten oxide semiconductors synthesis techniques have been reported, including electron beam evaporation [6,7], thermal or anodic oxidation [8,9], electrochemical deposition [10], chemical vapor deposition (CVD) [11,12], sputtering [13,14,15], pulsed laser deposition [16,17], sol-gel [18] as well as screen printing of thick films based on ultra-fine powder [19].

To date, with different synthesis techniques, tungsten oxides of various crystalline structures and morphologies ranging from zero-dimensional (nanoparticles and quantum dots), to 1D (nanorods, nanowires, nanofiber, nanobricks and nanotubes), 2D (nanosheets, nanoplates and nanolamella), and 3D (nanoflowers and nanospheres) [20] have been fabricated. The tungsten oxides-based gas sensors for the measurement of H_2_ [21], NO_2_ [18,22,23], NH_3_ [24,25,26,27], NO [22,27], H_2_S [28,29], CO [30,31] and organic gasses [32,33] such as acetone, methanol, ethanol and formaldehyde. Compared with bare tungsten trioxide (WO_3_) materials, the sensing properties have been significantly enhanced by the doping of other elements, functionalization of noble metal nanoparticles, or heterojunction formed with other semiconductors.

Since it was first reported as an *ammonia* sensor in 2000 [34], a pure tungsten trioxide-based gas sensor has demonstrated success in NH_3_ leak detection in both indoor and outdoor air quality monitoring [3,5]. However, these sensors normally operated at high temperatures ranging from 150 to 500 °C, which complicated the design requirements for temperature monitoring and control, as well as environmental safety. For example, Leng et al. reported that the optimal sensitivity occurred at 500 °C when exposed to 100 ppm NH_3_ [35]. Ji et al. reported recently that the sensitivity peaked at 350 °C when exposed to 100 ppm NH_3_ [36]. More recently, a great effort has been made to decrease the operating temperature, especially for wearable applications. As a result, ammonia detection around 150 °C was developed [37], as well as room temperature ammonia sensors by compositing carbon nanotubes [38], or hybridizing polyaniline [39], with tungsten oxide nanocrystals. Nevertheless, either the response time or recovery time was long, up to a few minutes. In order to have the sensing material return to initial conductance quickly after each cycle, a short pulse of annealing treatment at 250 °C was introduced.

Great advances have been achieved in designing and fabricating different dimensional WO_3_ materials. However, it is still challenging to achieve high-performance WO_3_ gas sensors. As an *n*-type semiconductor, its conductance increases when the chemisorbed oxygen species on the sensing material surface reacts with the adsorbed target gas molecules. Therefore, the material morphology and crystalline structure play a fundamental role in determining the sensing performances because the interaction occurs primarily on the surface and boundary. A quick, low-cost and environmental green device prototyping method is highly needed to optimize process parameters with a controllable approach and to explore the relationship between the featured morphologies and sensing performances.

The device performances reported to date have been evaluated for WO_3_ gas sensors fabricated based on a combination of different processing and design parameters, such as synthesis method, material composition, device dimension and electrode design. Hence, it is difficult to compare and optimize their performances. In this paper, we report on the simple post-annealing-free hot-filament CVD method to prepare tungsten oxide samples where tungsten oxides are grown by direct thermal evaporation of the tungsten filament under medium vacuum. Then, the evolution of the material compositions, crystalline structures and surface morphologies at different substrate temperatures is studied using scanning electron microscopy (SEM), energy dispersive x-ray spectroscopy (EDS), Raman spectroscopy, and X-ray diffraction (XRD) techniques. The gas-sensing properties based on the materials synthesized are examined systematically and compared with pure counterparts. Finally, a low-cost, high-performance gas sensor operating at room temperature is demonstrated for monitoring air pollutants such as NH_3_, CO and CH_4_.

## 2. Experimental

This simple and cost-effective synthesis technique was different from the method used in our previous work [40,41,42,43]. In the present work, the tungsten oxide composites were synthesized using a simple hot-filament CVD technique where the tungsten filament itself acted as a precursor for tungsten oxide, without using any catalyst or other tungsten-containing compound precursors. AlN ceramic disks and Si wafers were used as substrates which were ultrasonically washed in a methanol solution for 5 min, then rinsed with deionized water and finally dried before being loaded into the chamber. Then, the chamber was pumped down by using a rotary vacuum pump to reach a dynamic equilibrium of 200 mTorr without feeding any additional gases into the chamber. To prevent the oil molecules from backstreaming into the vacuum chamber, a special backstream trap/valve was installed in front of the turbo molecular pump that was located between the chamber and the mechanical pump in our system, although there was no need to operate this turbo molecular pump to achieve a pressure of 200 mTorr. To minimize any possible effects caused by the water molecules in the air, we used the dry air to purge the system after it was pumped down with the turbo pump before we used the mechanical pump to achieve the desired pressure. The substrate temperature was controlled within the range of 400 to 1000 °C simply by adjusting the distance between the hot filament and the substrate. An electrical current of ~20 A was applied to the hot filament. Once the hot-filament was heated up to 1500–2000 °C, the partial pressure of the residual gases balanced the partial pressures of the vaporized tungsten oxides and tungsten metal, while the substrate temperature provided the thermal energy required for the crystallization on the substrate. The synthesis process lasted for approximately 30 min, and no post-annealing process was applied to the synthesized samples. The estimated average deposition rate was around 50 nm/sec, but the total thickness varied slightly due to the variation in layer densities from different nanostructures. The actual thin film thickness was calibrated after the deposition.

## 3. Results and Discussions

### 3.1. SEM Measurements

**Figure 1:**
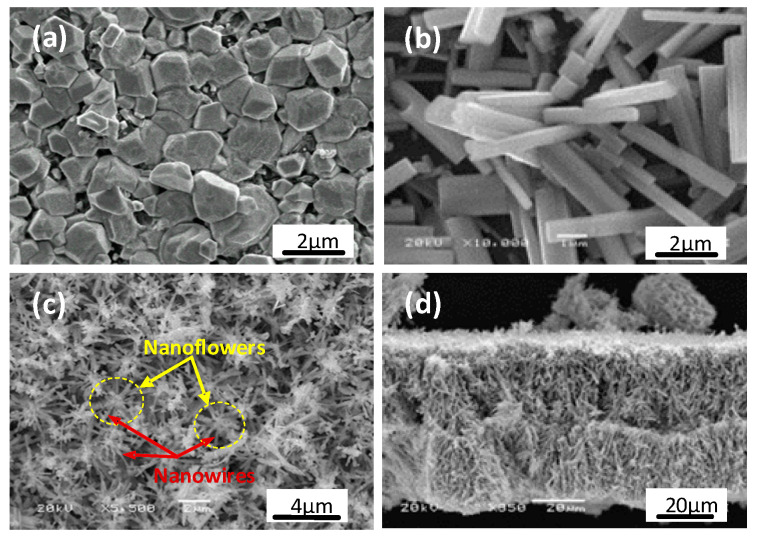
SEM images of the tungsten oxide membranes prepared at different substrate temperatures for 30 min. (**a**) Nanoparticles prepared at 1000 °C; (**b**) Nanorods at 700 °C; (**c**) Nanoflowers at 400 °C; and (**d**) The side view of the nanoflower sample prepared at 400 °C.

In general, WO_3_ is a complicated material because of its monoclinic, triclinic, tetragonal, orthorhombic, cubic, and hexagonal structures for pure and oxygen-deficient WO_3_. In addition, the synthesized thin films possess different micro- and nanostructures which depend upon the substrate temperature and oxygen partial pressure in the chamber. Detailed analyses of these polycrystalline structures and defects of WO_3_-x have been carried out by other groups [44,45,46,47,48]. Figure 1 shows SEM images of the tungsten oxide materials synthesized with substrate temperatures at approximately 1000, 700 and 400 °C. It is expected that the oxygen concentration will be low if the substrate temperature is high, and vice versa. In general, the tungsten oxide membrane prepared at 1000 °C appears grey-white in color with tiny particles of sizes varying from sub-micro to few micrometers in diameter, as shown in Figure 1a. The shaped edges of each particle indicate a good crystalline structure.

The synthesis at 700 °C substrate temperature yields different tungsten oxide composites, as indicated by its dark blue and dark black color. As shown in Figure 1b, randomly distributed over the substrate surface, are short and well-shaped rods, with an average diameter of ~0.8 µm and a length of ~5 µm. After a further decrease in the substrate temperature to 400 °C, the obtained tungsten oxide composites become completely dark black. As shown in Figure 1c, the membrane is composed of a large number of micrometer-sized flowers, and each flower is made of 10–12 nanowires. The average diameter and length of each wire are around 100 nm and 3 µm, respectively. The thickness estimated from the side view as shown in Figure 1d is around 60 µm. For other samples prepared above 400 °C, the measured thicknesses are slightly less than 60 µm, because the formed structures are more compact when crystallized at higher temperatures. Here, it should be mentioned that a structural transition would unavoidably occur at the film–substrate interface because of the shear stress caused by the misfit in lattices. When the film becomes thicker than its critical thickness, the membrane can easily be separated from the substrate. As shown in Figure 1d, the highly porous structure of the tungsten oxide membrane is expected to enhance gas molecule adsorption and to improve material-sensing properties. The free-standing membrane is then transferred onto a pair of Cu supports for the deposition of metal electrodes, material characterization and performance test.

### 3.2. EDS and XRD Measurements

**Figure 2:**
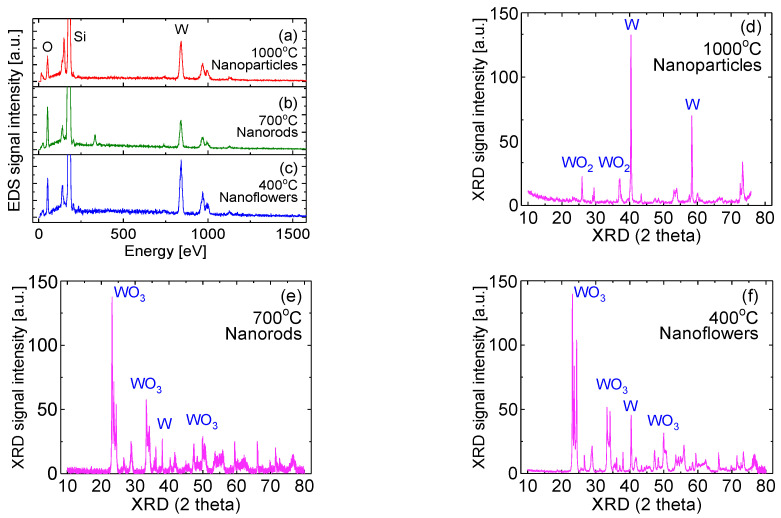
EDS of tungsten oxide membranes prepared at (**a**) 1000 °C, (**b**) 700 °C and (**c**) 400 °C, and XRD spectrum of the membrane prepared at (**d**) 1000 °C, (**e**) 700 °C and (**f**) 400 °C.

Figure 2a–c shows typical energy dispersive spectral measurements of the membranes synthesized at these three typical temperatures. In Figure 2a–c, the EDS signal from tungsten is higher than that of oxygen but the reverse is lower in Figure 2b. The ratio of the number of oxygen atoms to tungsten atoms is 1.8, 2.4, and 2 for NPs, NRs, and NFs, respectively. Clearly, the ratio of oxygen to tungsten atoms does not increase linearly as the crystallizing temperature increases. The incorporation of oxygen atoms relies on its concentration in the chamber and the competition between the trapping of oxygen atoms on the surface to form tungsten oxides and the desorption of O-containing species from the growth surface. Since the dissociation energy of O_2_ is high, the collision dissociation of O_2_ would not be large. Generally, there is a relatively small number of oxygen molecules inside the chamber at a high temperature if the gas pressure remains the same. The main mechanism of losing oxygen-containing species from the growth surface is thermal desorption. As a result, the deposition at a high temperature yields a low oxygen content of the tungsten oxide membrane. The experimental data from XRD measurements explain the different surface morphologies and material properties related to relative tungsten or oxygen contents inside the membranes. As shown in Figure 2d–f, WO_3_ dominates the sample prepared at a temperature of 700 °C and below, whereas W and WO_2_ normally dominate the sample prepared at 1000 °C.

Interestingly, EDS data in Figure 2c show that the number of oxygen atoms is only two times that of tungsten atoms. This phenomenon seems contradictory to the fact that the high temperature is one of the reasons for losing oxygen. However, the formation of tungsten oxide membrane is much more complicated. Normally, the obtained tungsten oxide composites have a mixture of WO_3_, WO_2_, and W elements, but polycrystalline WO_3_ dominates the membranes prepared at the substrate temperature of 400 to 700 °C. It is found that the metal W composition in the NF sample is significantly high. Consequently, this is confirmed with its low O/W ratio in EDS measurements, and with its high conductivity in I-V measurements.

### 3.3. Raman Spectral Measurements

**Figure 3:**
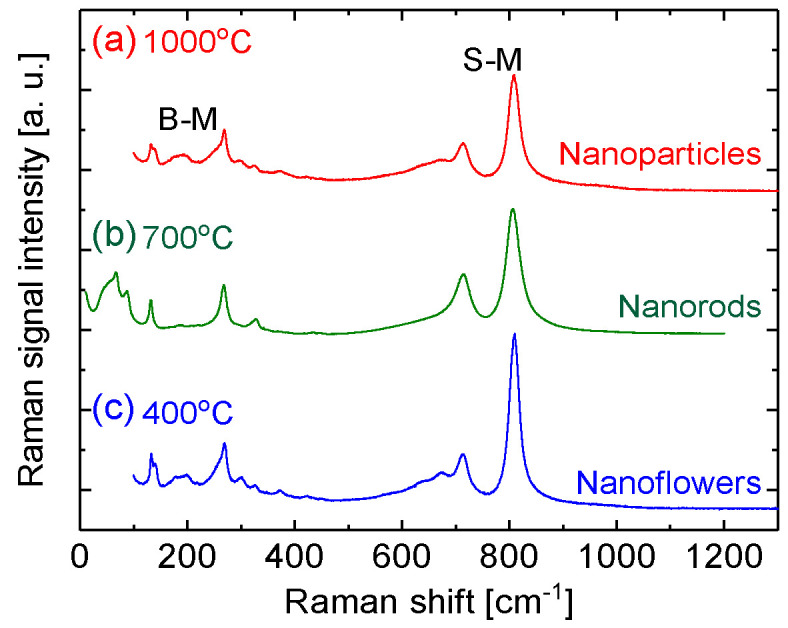
Raman spectra of the tungsten oxide membranes prepared at (**a**) 1000 °C, (**b**) 700 °C, and (**c**) 400 °C.

The phases and modes of tungsten oxide membranes were also examined using Raman scattering spectra with the 514.5 nm spectral line of an Ar ion laser. Figure 3 shows typical results in the range from 50 to 1500 cm^−1^ from the samples synthesized at substrate temperatures of 1000, 700 and 400 °C. According to previous work [1,41], the bands situated at around 700 and 800 cm^−1^ were assigned to W-O stretching modes (S-M), whereas the bands situated at around 130 and 270 cm^−1^ were associated with W-O bending modes (B-M) of monoclinic WO_3_.

As shown in Figure 3, the narrow Raman spectral lines from the three samples indicate their good crystalline structures, which agrees with the SEM observations shown in Figure 1 where each nanostructure has shaped edges. It is found that in bending modes, the spectral line ratio between the higher and lower wavenumber of the line increases with an increase in the substrate temperature at which the film is synthesized. However, no similar phenomenon is observed from the stretching modes. Previous work indicated that spectral lines were blue shifts following an increase in the substrate temperature during synthesizing [43]. In general, the blue shift in the Raman peaks should result from the change in crystal symmetry. It was also possibly due to the high stress of the samples. However, no clear shifts in the Raman peaks are observed in the present cases, which suggests that the stresses for all the synthesized structures are small.

### 3.4. Fabrications of Prototypes

**Figure 4:**
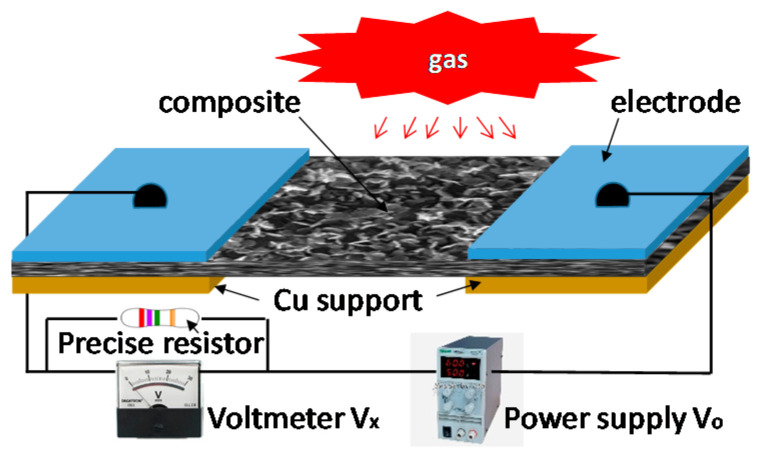
Schematic of the tungsten oxide based gas sensor (not drawn to scale). The circuit includes the sensor, adjustable voltage power supply *V_o_*, precise resistor *R_p_* and voltmeter *V_x_*.

After basic characterizations, these membranes of approximately 10 × 4 mm^2^ in size were used to develop room-temperature gas-sensing devices. As shown in Figure 4, approximately 80–100-nm-thick Au electrodes were directly deposited onto the two ends of the as-grown tungsten oxide membrane using plasma sputtering deposition technique without any annealing before or after the deposition. The spacing between the two electrodes was 1.5 mm, which was defined without employing any micro-machining techniques but a metal stencil. The free-standing, NF-based gas sensor was expected to have a higher sensitivity since its substrate has been removed so both sides of the membrane can be exposed to the target gas. The sensor fabrication was simple and cost-effective, resulting in an exposure area of around 6 mm^2^ for NP- and NR-based prototypes but around 12 mm^2^ for the NF-based prototype.

### 3.5. Electrical Properties and Bias Effect

**Figure 5:**
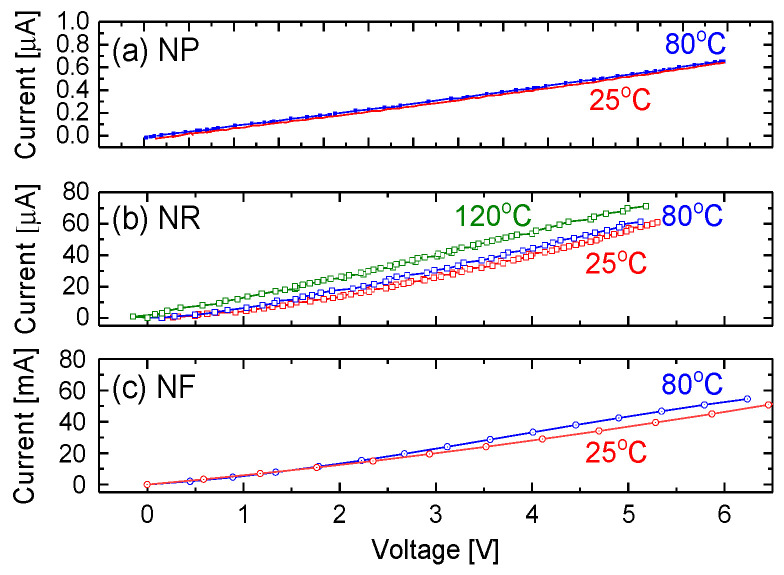
The typical I-V curves of (**a**) nanoparticle (NP)-, (**b**) nanorod (NR)-, and (**c**) nanoflower (NF)-based prototypes operated at different temperatures.

The electrical properties of the sensing prototype were characterized by two HP34401 electrical multimeters by connecting the fabricated device in series with a power supply (Agilent 6268B). Figure 5 shows the typical current-voltage (I-V) curves of the NP, NR and NF based prototypes, respectively, from room temperature to 120 °C. Good ohmic contact behaviors are indicated by nearly linear current-voltage curves. A slight increase in current with applied bias voltage is observed, due to the increase in the carrier drift velocity. As seen in Figure 5c, the large conductivity of the NF-based device mainly results from its higher concentration of tungsten. An increase in sensor operating temperature does not greatly affect electric properties. At 25 °C, the estimated resistances were 23 MΩ, 110 kΩ and 150 Ω for NPs, NRs and NFs, respectively, when biased at 3 V.

The sensitivity characteristics of a prototype exposed to a target gas were examined based on the measurement of the voltage drop across the precise resistor (*R_p_*) before (in ambient air) and after the sensor was exposed to the target gas. The *R_p_* value was chosen to be comparable with the resistance of the prototype. For example, *R_p_* = 100 Ω was used for the characterization of an NF-based device. The definition of the response (*α*) is based on a relative impedance change in the sensing material

α = (*R_o_* − *R_x_*)/*R_o_* (1)

where *R_o_* and *R_x_* are the impedances of the sensing material in ambient air and after exposure to the target gas. It has been found that the NP-based sensor exhibits a very poor response, likely due to the relatively small surface to volume ratio which does not provide sufficient area for gas adsorption and diffusion. Therefore, in the following sections, the focus will be on NR- and NF-based sensors.

**Figure 6:**
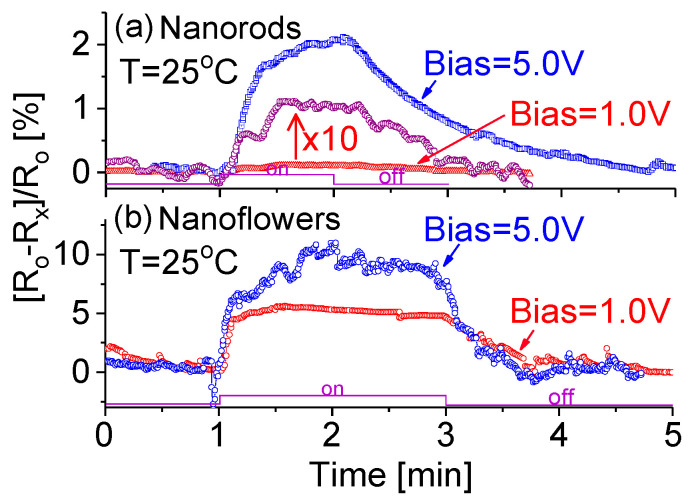
Responses of (**a**) NRs- and (**b**) NFs-based prototypes at room temperature to 1.2k ppm NH_3_ at biases of 1 and 5 V.

To test sensor selectivity, experiments were carried out with different target gases, such as CO, NH_3_ and CH_4_, using the gas sensor characterization system described in detail previously [49]. Briefly, the fabricated sensors were tested in a gas chamber of a dimension of 15 × 15 × 15 cm^3^ which contained a thermocouple for temperature monitoring, a heater for operating temperature setting, the target gas-in and -out feedthroughs with gas flow controllers, an adjustable bias voltage, and a voltage-current-resistor (*V*-*I*-*R*) electrical circuit. Since the NH_3_ sensitivity is about five times greater than CO, the present work will concentrate on NH_3_ and CH_4_ sensor performances at room temperature. Figure 6 shows the room temperature responses (*α*) of NR- and NF-based prototypes exposed to ammonia gas at biases of 1 and 5 V. Although a higher bias yields a larger response, it does not affect the rise time, which is much shorter than the recovery time. As shown in Figure 6a, the NR-based prototype exhibits a clean response. Experimental data also indicate that at the same applied bias, the NF-based prototype has a larger response strength with *α* = ~10% at 5 V bias, and *α* = ~5% at 1 V bias, as shown in Figure 6b. Furthermore, either rise time or recovery time is significantly shorter than that of the NR-based device. This can be directly attributed to the different electric and electronic properties of the nanocomposites under investigation.

### 3.6. Effect of Gas Concentration

**Figure 7:**
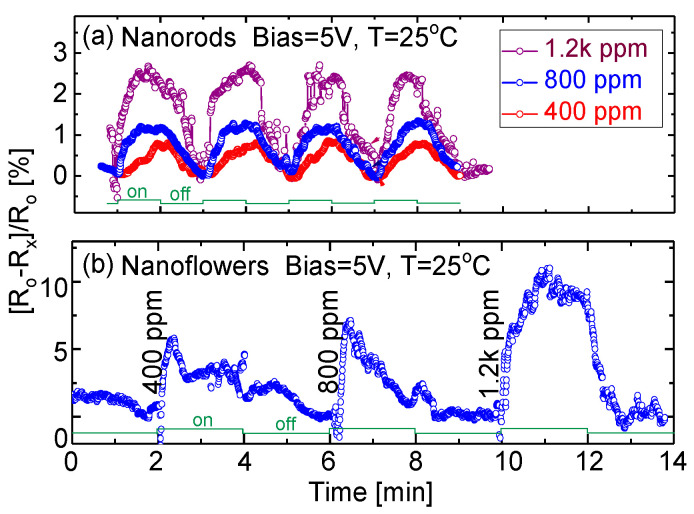
Responses of the prototypes based on (**a**) NRs, and (**b**) NFs towards NH_3_ gas.

Since the fabricated prototype gave a higher response when biased with a DC voltage, a 5 V bias was used for the following cases except when specified. Typical room temperature responses of the prototypical sensing devices exposed to different NH_3_ concentrations are shown in Figure 7. When the prototype is exposed to the target gas, the response signal of the fabricated sensor quickly increases and reaches its maximum. When the target gas input valve is turned off, the response signal decreases and then gradually recovers to its original state. Variation in the response closely follows the on–off cycles of the gas flow valve. Such a change in material impedance could be directly attributed to the adsorption of the target gas molecules on the sensing material surface. A relatively strong noise signal was due to the simple circuit (Figure 4) and a highly humid environment. The noise signal level would be greatly reduced if the prototypes were tested in a dry air environment using coaxial cables integrated with an operational amplifier-based active filter circuit.

It is also noticed from Figure 7b that the NF-based device has a shorter response time, around 10 s, whereas it is around 50 s for the NR-based device. Response and recovery times are calculated as the time duration from 10% to 90% of the full response of the device, or vice versa. In general, a quick response time is closely related to high sensitivity. However, the stability of the NF-based device seems poor. The noise signal is relatively strong, yielding a low signal-to-noise ratio. This phenomenon might be attributed to a different mechanism related to the adsorption and desorption of target gas molecules on the surface of sensing material. Due to the large active sites and high surface-to-volume ratio, the porous-structure-based gas sensor appears to have a fast response, leading to an increase in sensitivity. In contrast, the recovery speed is not comparably fast, because it takes a longer time to recover to its original state after the chamber gas environment has been switched from the target gases to the air. The relatively slow desorption of gas molecules is in good agreement with the porous SnO_2_-based gas sensor [50].

**Figure 8:**
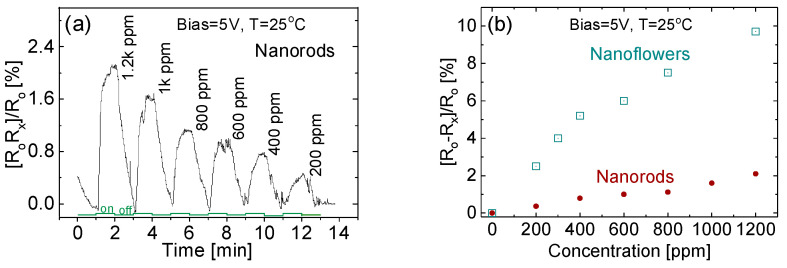
(**a**) The response signals from the NR-based prototype, and (**b**) the quasi-linear responses of NF- and NR-based prototypes to NH_3_ of different concentrations.

The responses of the prototypes when exposed to NH_3_ of different concentrations from 200 to 1.2k ppm have been tested, and Figure 8a shows the signals for the NR-based prototype. As shown in Figure 8b, response strengths from both NR- and NF-based prototypes appear to have a quasi-linear relationship with NH_3_ concentrations, from which we can estimate the sensitivity *β* at around 2 × 10^−3^ per ppm for the NR-based device and around 8 × 10^−^^3^ per ppm for the NF-based device.

Definition of the sensitivity (*β*) is based on the response-to-target gas concentration ratio

*β* = Δ*α*/Δ*N* (2)

where Δ*α* and Δ*N* are the changes in response strength and target gas concentration, respectively. The sensitivity *β* of the NF-based device is four times as large as that of the NR-based device. Possible reasons for this phenomenon may rely on three factors: (1) the surface to volume ratio; (2) the distinct compositions inside the membrane that affect device sensitivity; and (3) the strain lattice defects and conjunction effects that occur in the membrane because of the large number of nanowire–nanowire interphases and orientations. Previous theoretical and experimental data revealed that an extended line defect across wires could significantly affect electric properties. It is reasonable to speculate that the observed performance in the present case is the result of a superposition of the factors listed above.

### 3.7. Temperature Effect

**Figure 9:**
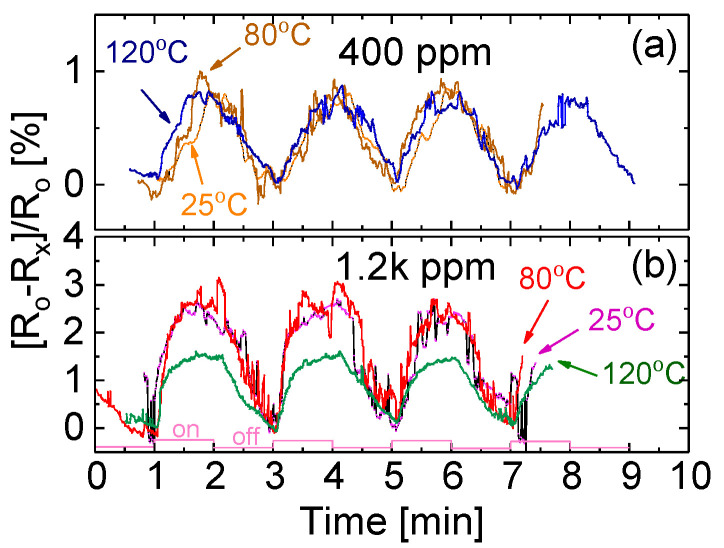
Temperature effect on responses of the NR-based prototype exposed to (**a**) 400 ppm and (**b**) 1.2k ppm NH_3_ when cycled at 25, 80 and 120 °C.

It has been reported by many research groups that the variation in temperature would significantly affect the device sensitivity and response [1,3]. However, it is found in the present investigation that the variation in operating temperature may only slightly affect the performance of the NR-based prototype exposed to NH_3_ gas, as shown in Figure 9. As shown in Figure 7a, at 25 °C the response time is around 30 s and the recovery time is around 60 s. Figure 9 shows that, after the temperature increases from 25 to 80, and then to 120 °C, both the response time and the recovery time remain nearly unchanged. However, the response signal strength for the NR-based device exposed to NH_3_ gas with a concentration of 1.2k ppm is slightly enhanced from 2.2% to 2.8% with an increase in operating temperature from 25 to 80 °C and then drops down to 1.5% following a further increase to 120 °C.

**Figure 10:**
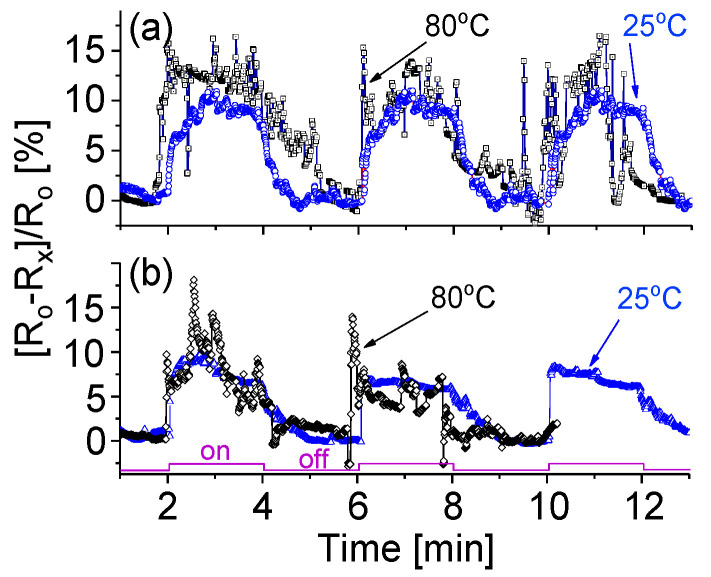
Responses of NFs-based prototype when cycled with (**a**) 1.2k ppm NH_3_ and (**b**) 1.2k ppm CH_4_ at 25 and 80 °C.

Relatively, the NF device has a better response or sensitivity to NH_3_ target gas than that of the NR device operating at either room or medium temperatures. As shown in Figure 10a, following an increase in the operating temperature from 25 to 80 °C, the response strength increases from *α* = 11% to *α* = 14%. Furthermore, a good response time of less than 10 s is obtained. However, the noise signal appears relatively strong. A similar phenomenon is also observed from the NF-based device exposed to CH_4_ target gas, as shown in Figure 10b. From the obtained experimental data above, four conclusions can be drawn. (1) A simple way to enhance the sensitivity is to employ a slightly higher bias and operating temperature but the too-high temperature would cause an intense noise signal. (2) The NF-based device appears to have a higher sensitivity to the target gases but a correspondingly strong noise in the output. (3) The NF-based sensor has a shorter response time than that of the NR-based device, which is nearly independent of the bias magnitude and operating temperature. (4) The fabricated tungsten oxide-based sensors have a higher sensitivity towards NH_3_ than that towards CH_4_ gas.

The results show that the variation in temperature could not significantly affect device sensitivity, response and recovery. We believe that this phenomenon could be attributed to the fact that the present nanostructured materials have good thermal conductibility. Therefore, the variation in temperature does not greatly affect electric properties. This feature has been confirmed with experimental data shown in Figure 5. The obtained results related to temperature effects on responses of the fabricated prototype as shown in Figure 9 and Figure 10 are also in good agreement with the results shown in Figure 5.

A comparison of the properties is listed in Table 1 for pristine tungsten oxide-based NH_3_ gas sensors reported recently [5,25,26,35,36,37]. To date, all of the reported NH_3_ resistive gas sensors using undoped tungsten oxides worked only above room temperature. The room temperature detection of NH_3_ was not reported unless the tungsten oxide sensor was doped with other elements [27] or formed by using the hybrid nanocomposite with other functional materials [38,39]. The gas sensor presented by this work shows a decent response to NH_3_ target gas at room temperature. Moreover, it has a fast rise and decay time.

Several factors might affect the accuracy in the response measurement such as gas flow control, humidity, power supply stability, electrical noises, etc. One major factor is the humidity effect since the characterizations of sensing capabilities were carried out in an ambient environment with high relative humidity. The local climate is tropical marine with an average percentage of humidity up to 75.0%. If dry air was used, higher sensitivity and a more stable signal could be obtained. Furthermore, if coaxial cables were used with an operational amplifier-based active filter to replace the current simple circuit, as shown in Figure 4, the noise signal would be greatly reduced. After making the comparison of all data from repeated experimental measurements, we can conclude that the relative error should be less than 17% in all reported results.

## 4. Conclusions

The simple post-annealing-free hot filament CVD method has been used to effectively prepare crystalline tungsten oxides. Compared with the other synthesizing methods, this simple approach offers some advantages such as its easy implementation, cost effectiveness, and the avoidance of harmful agents. The controllable compositions and morphologies, including NPs, NRs and NFs, have been obtained at a relatively high deposition rate by varying the substrate temperature. Based on these as-grown tungsten oxide membranes, simple, low-cost gas-sensing prototypes have been designed, fabricated, and tested. The NF-based gas sensor gives the best performance, followed by the NR-based device and lastly the NP device. When operated at room temperature, besides high sensitivity and fast response time, the NF-based gas sensor offers the additional advantage of good repeatability and stable baseline. Several ways have been suggested to further enhance the gas-sensing sensitivity. Besides the functionalization of the active layer surface with noble metal nanoparticles, it is found that in general, hybrid nanocomposite or multistructure-based sensors have a higher sensitivity than a conventional device constructed solely from one material when tested under identical experimental conditions, suggesting a synergistic effect between the two components. Overall, such a simple fabrication method can be further use to optimize the sensor performance by fine tuning processing parameters such as oxygen content inside the vacuum chamber, substrate temperatures for other morphologies like nanotubes, and thermal annealing processes. In addition, this method can also be modified for doping with other elements, or functionalization with noble metal particles, etc., to further optimize the material properties for sensing different target gases.

## Figures and Tables

**Table 1:** Comparison of NH_3_ gas sensor properties based on tungsten oxides reported recently in the literature.

Materials	NH_3_ (ppm)	Temp. (°C)	Response Formula ^#^	Response	Reference
Undoped WO_3_	400	450	*S* = *R_g_*/*R_a_*	1.04	[25]
Undoped WO_3_	100	400	*S* = *R_g_*/*R_a_*	9.8	[26]
Undoped WO_3_	30	350	*S* = *R_g_*/*R_a_*	5.40	[5]
Undoped WO_3_	100	500	*S* = *R_g_*/*R_a_*	5.5	[35]
Undoped WO_3_	100	350	*S* = *R_g_*/*R_a_*	36.3	[36]
Undoped WO_3_	120	150	*S* = (*R_a_ − R_g_*) × 100/*R_a_*	73%	[37]
Pt-WO_3_	200	125	*S* = *R_g_*/*R_a_*	13.61	[27]
PANI/WO_3_	100	RT	*S* = *R_g_*/*R_a_*	20.1	[39]
1.0 wt % CNT/WO_3_	10	RT	*S* = (*R_a_ − R_g_*) × 100/*R_a_*	6.8%	[38]
Undoped WO_3_	200	RT	*S* = (*R_a_ − R_g_*) × 100/*R_a_*	0.4%	Present work

*^#^ R_g_*: sensor impedance in target gas; *R_a_*: sensor impedance in ambient air.
